# Matrix Metalloproteinase Expressions Play Important role in Prediction of Ovarian Cancer Outcome

**DOI:** 10.1038/s41598-019-47871-5

**Published:** 2019-08-12

**Authors:** Shujie Wang, Jia Jia, Dongyan Liu, Meng Wang, Zhen Wang, Xueling Li, Hongzhi Wang, Yong Rui, Zhirong Liu, Wei Guo, Jinfu Nie, Haiming Dai

**Affiliations:** 1 Anhui Province Key Laboratory of Medical Physics and Technology, Center of Medical Physics and Technology, Hefei Institutes of Physical Science, Chinese Academy of Sciences, Hefei, Anhui China; 2 Hefei Cancer Hospital, Chinese Academy of Sciences, Hefei, Anhui China; 3 Hefei Institute of Stem Cell and Regenerative Medicine, Guangzhou Institutes of Biomedicine and Health, Chinese Academy of Sciences, Hefei, China

**Keywords:** Cancer, Computational biology and bioinformatics

## Abstract

Ovarian cancer has a high death rate and is often not detected until late stages. While some studies found high expressions of MMPs correlated with cancer invasion, metastasis, and poor prognosis, however, several other studies indicated MMPs might inhibit cancer rather than promote cancer in certain situations. Thus, the role of different MMPs in different cancer types needs a systematic re-evaluation. In this study, we used RNA-Seq and corresponding clinical data downloaded from TCGA and analyzed the correlations between MMP expressions and the clinicopathologic characteristics and outcome in ovarian serous cystadenocarcinoma (OSC) patients. Among the MMPs investigated, MMP-3 was significantly increased in high-grade and high-stage tumors compared with low-grade and low-stage ones. Using univariate analysis and multivariate Cox model, high expressions of MMP-19 and -20 were found to associate with poor overall survival independent of clinicopathologic characteristics. Moreover, using *in vitro* studies, we found ovarian cancer cell lines with higher MMP-19 and -20 protein expressing levels were associated with anti-cancer drugs resistance, while knockdown of MMP-19 or -20 increased ovarian cancer cell sensitivities to several clinical using chemotherapy agents. Finally, knockdown of MMP-19 or -20 also decreased the invasion abilities of several ovarian cancer cell lines. These *in vitro* studies provided potential mechanisms of high MMP-19 and -20 expressions in the poor prognosis of ovarian cancer.

## Introduction

Ovarian cancer, which was frequently not detected until later stages, has a high death rate among gynecological cancers^1^. According to the 2014 statistics of the National Cancer Institute (NCI), ovarian cancer is the fifth leading cause of cancer death in women^2^. On the other hand, ovarian cancer is characterized by its widespread metastasis^3^.

Matrix metalloproteinases (MMPs) are a family of proteolytic enzymes^4^, which are often calcium-dependent and zinc-containing^5^, which could degrade all kinds of extracellular proteins. At least two groups of the extracellular proteins have been reported to play important roles in cancer metastasis and invasion. The first group includes some of the extracellular matrix components, such as fibronectins, gelatins, collagens and so on, which could prevent the cancer cells from moving freely into the nearby tissues, vessels and lyphatics^6–8^. The second group includes some cell surface receptors, ligands, and chemokines and cytokines^9,10^, which play important roles in cancer cell migration, proliferation or apoptosis through different cell signaling pathways. Because of the abilities of MMPs in degrading these extracellular proteins, high expressions of MMPs have been widely reported to be related to cancer metastasis, progression, and poor prognosis^11,12^. For example, MMP-19 was highly expressed in astroglial tumors and could facilitate the invasion of glioma cells through brain extracellular matrix components^13^. MMP-20 showed an important role in the progression of esophageal cancer^14^. In addition, MMP-14 was reported to be associated with the invasion and metastasis of ovarian carcinoma^15^. More than the degradation of extracellular proteins, some MMPs regulate tumor invasion through other mechanisms, for example, MMP-2 as well as MMP-9 could activate TGF-β to promote tumor invasion^16^.

On another hand, although most studies have found poor prognosis correlated with the high expressions of MMPs^15,17–22^, a few other studies have found certain MMPs could inhibit cancer growth rather than promote it under certain situations^23–25^, suggesting that different MMPs have different roles. Moreover, while several MMP inhibitors have been developed^26,27^, however, there are still no effective MMP inhibitors to treat cancer today. More importantly, studies have also suggested that some MMP inhibitors might promote cancer progression instead of inhibition^27^, which limited the usage of MMP inhibitors in the clinic. Therefore, to evaluate the significance of MMP expressions in the prediction of cancer prognosis, systematic studies of the relationships between each MMP and certain cancers were needed.

In this study, we used the RNA expression data from The Cancer Genome Atlas (TCGA) to conduct a systematic investigation of the relationships between each MMP members and the clinical characteristics in ovarian serous cystadenocarcinoma (OSC). Our study not only found some MMPs were correlated with the clinicopathologic characteristics in OSC patients, but also found high expressions of MMP-19 as well as MMP-20 predicted poor outcome. Further studies found MMP-19 and MMP-20 high expressions could cause drug resistance and cancer invasion, providing potential molecular mechanisms for the poor prognosis caused by high MMP-19 and MMP-20 expressions.

## Results

### Patient clinical characteristics

293 ovarian cancer patients from TCGA with clinical and gene expression data were analyzed in this study. The clinical characteristics were shown in Table 1. The median age of the patient cohort was 58 years (range 30–87 years). Among these patients, about 89% were Caucasian, 6% were African or African American, and 4% were Asian. The proportion of American Indian, Alaska Native, or Native Hawaiian and other Pacific Islander was less than 1%. The proportions of Stage I, II, III, and IV of these patients were 0%, 6%, 82%, and 12%, respectively. The overall grades for these patients were: 11% at Grade 1 and 2, 89% at Grade 3, and less than 1% at Grade 4. Right, left, and bilateral for anatomic neoplasm subdivisions were 13%, 12% and 75%, respectively. The median time to last follow up was 28.0 months with the range of 0–183 months.

**Table 1:** Clinical characteristics of 293 patients with OSC of TCGA cohort.

Characteristic	Total	%
**Age at diagnosis (Median age (range))**	58 (30–87)	
Race		
American Indian or Alaska Native	2	0.7
Caucasian	250	88.7
Native Hawaiian or other Pacific Islander	1	0.4
Asian	11	3.9
African or African American	18	6.4
Stage		
I	0	0.0
II	17	5.8
III	238	81.8
IV	36	12.4
Grade		
1	1	0.3
2	30	10.5
3	255	88.8
4	1	0.3
anatomic neoplasm subdivision		
Right	36	13.0
Left	33	11.9
Bilateral	208	75.1

### Relationship between MMPs expressions and clinicopathologic characteristics

**Figure 1:**
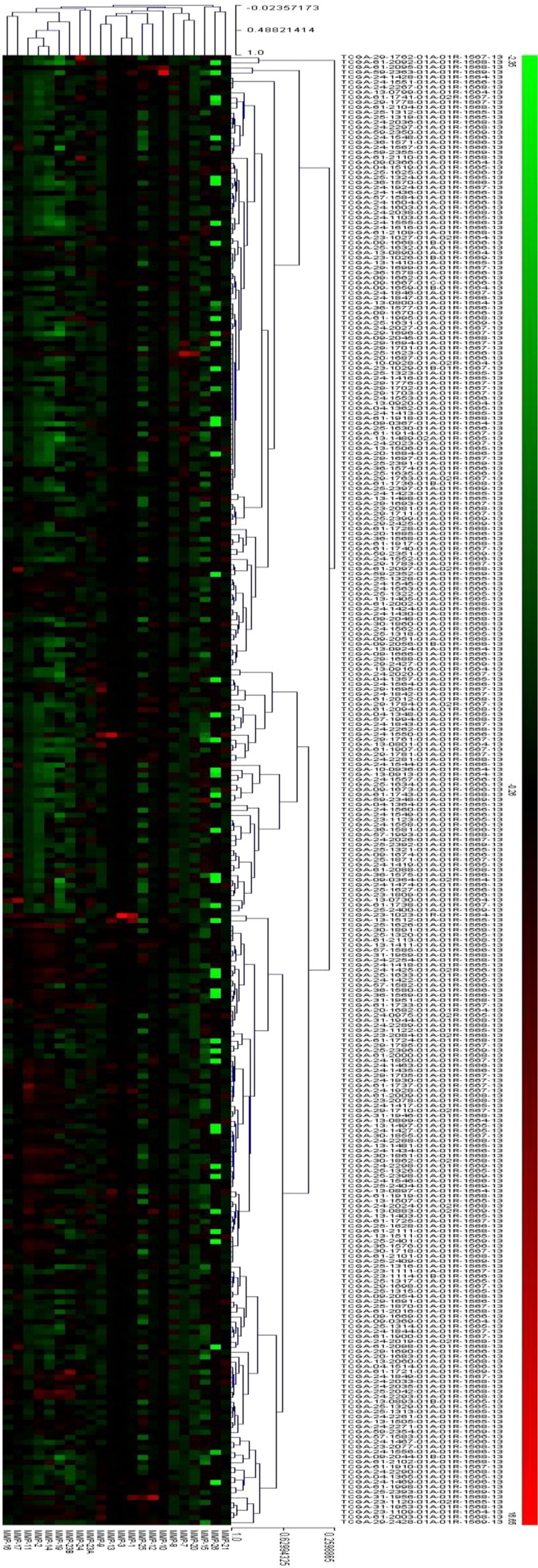
Expression profiles of MMPs in 293 OSC patients. Color changes represent varied MMPs expression levels.

We first analyzed the expression profile of 22 MMPs in the above 293 OSC patients. As shown in Fig. 1, the mRNA expression levels of different MMPs varied among the patient samples. Generally, MMP-2, MMP-7, MMP-11 and MMP-14 were expressed at higher levels than the other MMPs in most of the patient samples.

Previous studies have shown that some MMP expression levels correlate with clinicopathologic characteristics, for example, MMP-1, MMP-9, MMP-12 and MMP-15 expressed higher in grade 3 than in grade 1 and 2 breast tumors^28^. To further explore whether the MMP expression levels correlate with clinicopathologic status in OSC, we systematically analyzed the correlations between them, using the Mann-Whitney U test. As shown in Table 2, upon this analysis, we found mRNA levels of MMP-3 and MMP-25 were significantly higher in stage III and IV than stage II cancers (p < 0.05), while the other MMPs did not show significant differences between different stages. Moreover, Levels of MMP-3 and MMP-16 were significantly increased in higher grade (grades 3 and 4) compared with lower grade (grades 1 and 2) tumors (p < 0.05). Compare bilateral neoplasm subdivision group to one side neoplasm subdivision group, only levels of MMP-7 and MMP-9 showed significant differences (p < 0.05).

**Table 2:** Relationship between MMPs expression levels and clinicopathologic characteristics.

	MMP-1	MMP-2	MMP-3	MMP-7	MMP-8	MMP-9	MMP-10	MMP-11	MMP-12	MMP-13	MMP-14	MMP-15	MMP-16	MMP-17	MMP-19	MMP-20	MMP-21	MMP-23A	MMP-23B	MMP-24	MMP-25	MMP-26
**Stage**																						
II	3.042	43.041	0.340	79.901	0.090	36.302	2.369	25.823	5.117	0.724	71.659	18.194	0.106	2.240	5.633	0.034	0.076	0.058	0.848	3.813	1.341	0.038
III + IV	4.507	83.258	0.441	123.358	0.102	27.973	4.991	97.223	3.233	3.027	74.706	18.415	0.133	1.117	7.175	0.093	0.059	0.056	1.208	6.183	1.052	0.011
*P* value	NS	NS	0.037	NS	NS	NS	NS	NS	NS	NS	NS	NS	NS	NS	NS	NS	NS	NS	NS	NS	0.048	NS
**anatomic neoplasm subdivision**																						
Left or right	6.883	77.045	1.290	90.036	0.115	39.357	2.883	76.795	4.523	4.521	79.841	16.511	0.152	0.871	6.809	0.035	0.059	0.054	1.211	5.014	1.114	0.011
Bilateral	3.687	81.827	0.151	131.938	0.096	24.647	5.689	94.754	2.933	2.267	71.847	18.664	0.125	1.306	7.162	0.111	0.063	0.057	1.228	6.520	1.048	0.014
*P* value	NS	NS	NS	0.036	NS	0.032	NS	NS	NS	NS	NS	NS	NS	NS	NS	NS	NS	NS	NS	NS	NS	NS
**Grade**																						
1 + 2	3.764	93.325	0.410	141.643	0.161	41.070	9.558	99.666	5.751	1.879	78.207	16.427	0.130	2.143	6.826	0.042	0.094	0.044	1.538	6.678	1.170	0.020
3 + 4	4.533	80.773	0.445	112.884	0.095	27.422	4.330	92.433	3.099	3.018	74.924	18.550	0.132	1.074	7.162	0.091	0.056	0.059	1.161	5.792	1.062	0.012
*P* value	NS	NS	0.034	NS	NS	NS	NS	NS	NS	NS	NS	NS	0.048	NS	NS	NS	NS	NS	NS	NS	NS	NS

^a^“mRNA” (messenger RNA); “MMP” (matrix metalloproteinase); “NS” (not significance).

^b^The Mann-Whitney U test was used.

### Relationship between expression levels of MMPs

**Figure 2:**
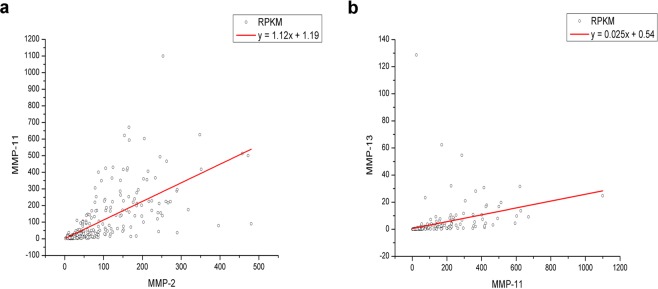
Stong correlations (|r| > 0.8) were found between the mRNA expressions of MMP-2/MMP-11 and MMP-11/MMP-13. The red solid line represents the result of linear fitting. Correlations of MMP-2/MMP-11 (**a**), and MMP-11/MMP-13 (**b**), from 293 OSC patients were shown respectively.

Previous studies have found that some MMP expressions are correlated, for example, significant correlations were found between MMP-2 and MMP-11, MMP-13 and MMP-14, and MMP-13 and MMP-14 in breast cancer^28^. To investigate correlations between expressions of different MMPs, the Spearman correlation analysis was used, and the results were shown in Table 3. Three different types of correlations were found between MMPs expression levels: strong correlations (|r| ≥ 0.8, also shown in Fig. 2) were found between MMP-2 and MMP-11, and between MMP-11 and MMP-13; medium correlations (0.5 ≤ |r| < 0.8) were found between 16 MMP pairs, including MMP-2/MMP-14, MMP-2/MMP-19, MMP-14/MMP-19 and so on; weak correlations (0.3 ≤ |r| < 0.5) were found between 41 pairs of MMPs.

**Table 3:** Correlation between mRNA levels of MMP family members in OSC patients.

	MMP-2	MMP-3	MMP-7	MMP-8	MMP-9	MMP-10	MMP-11	MMP-12	MMP-13	MMP-14	MMP-15	MMP-16	MMP-17	MMP-19	MMP-20	MMP-21	MMP-23A	MMP-23B	MMP-24	MMP-25	MMP-26
MMP-1	<0.001	<0.001	<0.001	<0.001	<0.001	<0.001	<0.001	<0.001	<0.001	<0.001	NS	<0.001	0.035	0.006	NS	0.009	NS	NS	NS	NS	NS
	r = 0.291	r = 0.524	r = 0.222	r = 0.399	r = 0.244	r = 0.438	r = 0.420	r = 0.455	r = 0.559	r = 0.261		r = 0.223	r = 0.123	r = 0.159		r = −0.153					
MMP-2	—	<0.001	0.002	<0.001	<0.001	<0.001	<0.001	0.003	<0.001	<0.001	NS	<0.001	<0.001	<0.001	0.028	NS	NS	<0.001	NS	0.035	0.003
		r = 0.525	r = 0.184	r = 0.319	r = 0.251	r = 0.213	r = 0.820	r = 0.170	r = 0.691	r = 0.764		r = 0.500	r = 0.427	r = 0.555	r = 0.129			r = 0.471		r = 0.123	r = −0.173
MMP-3		—	<0.001	<0.001	<0.001	<0.001	<0.001	<0.001	<0.001	<0.001	NS	<0.001	<0.001	<0.001	0.010	NS	NS	0.004	NS	NS	NS
			r = 0.257	r = 0.364	r = 0.228	r = 0.441	r = 0.603	r = 0.383	r = 0.635	r = 0.446		r = 0.267	r = 0.221	r = 0.312	r = 0.150			r = 0.167			
MMP-7			—	0.002	NS	<0.001	<0.001	0.006	<0.001	NS	NS	NS	NS	NS	<0.001	NS	NS	NS	NS	NS	NS
				r = 0.183		r = 0.219	r = 0.210	r = 0.161	r = 0.239						r = 0.556						
MMP-8				—	<0.001	<0.001	<0.001	<0.001	<0.001	<0.001	NS	0.030	0.012	<0.001	NS	NS	NS	NS	NS	NS	NS
					r = 0.459	r = 0.308	r = 0.330	r = 0.585	r = 0.403	r = 0.321		r = 0.127	r = 0.146	r = 0.270							
MMP-9					—	0.048	<0.001	<0.001	<0.001	<0.001	NS	NS	NS	<0.001	NS	NS	NS	NS	NS	<0.001	NS
						r = 0.115	r = 0.265	r = 0.594	r = 0.297	r = 0.327				r = 0.244						r = 0.297	
MMP-10						—	<0.001	<0.001	<0.001	0.041	NS	0.009	NS	NS	NS	NS	NS	NS	<0.001	NS	NS
							r = 0.247	r = 0.336	r = 0.383	r = 0.119		r = 0.152							R = −0.257		
MMP-11							—	<0.001	<0.001	<0.001	NS	<0.001	<0.001	<0.001	NS	NS	NS	<0.001	NS	NS	NS
								r = 0.224	r = 0.805	r = 0.702		r = 0.391	r = 0.331	r = 0.427				r = 0.322			
MMP-12								—	<0.001	0.001	NS	NS	NS	0.007	NS	NS	0.012	NS	<0.001	<0.001	NS
									r = 0.285	r = 0.195				r = 0.157			r = −0.146		R = −0.207	r = 0.216	
MMP-13									—	<0.001	NS	<0.001	<0.001	<0.001	0.039	NS	NS	0.002	NS	NS	0.043
										r = 0.594		r = 0.284	r = 0.234	r = 0.365	r = 0.121			r = 0.179			r = −0.118
MMP-14										—	NS	<0.001	<0.001	<0.001	NS	NS	0.022	<0.001	NS	0.023	NS
											r = 0.438	r = 0.531	r = 0.531	r = 0.516			r = 0.133	r = 0.389		r = 0.133	
MMP-15											—	NS	NS	NS	NS	NS	0.045	NS	NS	NS	NS
																	r = 0.117				
MMP-16												—	<0.001	<0.001	NS	0.028	0.006	<0.001	NS	NS	NS
													r = 0.303	r = 0.369		r = 0.129	r = 0.162	r = 0.324			
MMP-17													—	<0.001	NS	<0.001	0.028	<0.001	NS	NS	NS
														r = 0.282		r = 0.254	r = 0.129	r = 0.320			
MMP-19														—	NS	NS	<0.001	<0.001	0.028	<0.001	NS
																	r = 0.244	r = 0.378	r = 0.128	r = 0.317	
MMP-20															—	NS	NS	NS	NS	NS	NS
MMP-21																—	0.021	0.009	NS	0.039	NS
																	r = 0.135	r = 0.152		r = 0.121	
MMP-23A																	—	<0.001	NS	NS	NS
																		r = 0.498			
MMP-23B																		—	0.011	NS	0.039
																			r = 0.148		r = −0.121
MMP-24																			—	NS	NS
MMP-25																				—	NS
MMP-26																					—

^a^“mRNA”(messenger RNA); “MMP” (matrix metalloproteinase); “NS” (not significance).

^b^The Spearman correlation analysis was used.

### High MMP-19 and -20 expressions predict poor prognosis

Previous studies have found some MMPs correlated with overall survival in breast cancer patients^28^. To analyze the correlation between the expressions of MMPs and the overall survival in 265 OSC patients with complete clinic information, each MMP was treated as a continuous variable in a univariate cox model. Under this analysis (Table 4), high expressions of MMP-2, MMP-14, MMP-19 and MMP-20 were found to associate with poor overall survival. To find out whether these MMPs could serve as independent variables, the derived four MMPs were again analyzed with stage, grade, and anatomic neoplasm subdivision in a multivariate model. Only high expression of MMP-19 and MMP-20 were significantly associated with poor overall survival independent of the above clinicopathologic characteristics after this analysis (Table 5). Thus, high expressions of MMP-19 or MMP-20 could serve as independent factors to predict poor prognosis in OSC patients.

**Table 4:** Relationship between MMP expression and overall survival in OSC.

	All patients (n = 265)		
	*P* value	Hazard ratio	95% CI
MMP-1	0.119	0.975	0.945–1.006
MMP-2	0.044	1.002	1.000–1.004
MMP-3	0.385	0.883	0.667–1.169
MMP-7	0.749	1.000	0.999–1.001
MMP-8	0.293	1.546	0.686–3.482
MMP-9	0.704	0.999	0.993–1.004
MMP-10	0.149	0.978	0.949–1.008
MMP-11	0.735	1.000	0.999–1.001
MMP-12	0.101	0.972	0.940–1.006
MMP-13	0.835	1.002	0.986–1.018
MMP-14	**0.011**	1.004	1.001–1.007
MMP-15	0.112	0.988	0.972–1.003
MMP-16	0.068	2.616	0.932–7.343
MMP-17	0.988	0.999	0.934–1.070
MMP-19	**0.001**	1.091	1.036–1.148
MMP-20	**0.002**	1.692	1.217–2.353
MMP-21	0.525	1.548	0.403–5.945
MMP-23A	0.725	1.348	0.254–7.150
MMP-23B	0.344	1.084	0.917–1.282
MMP-24	0.671	0.995	0.974–1.017
MMP-25	0.159	0.797	0.581–1.093
MMP-26	0.871	1.498	0.012–194.715

^a^“MMP” (matrix metalloproteinase); “CI” (confidence interval).

^b^Data were analyzed using a univariate cox model.

**Table 5:** Relationship between MMP-19 as well as MMP-20 expression and overall survival in ovarian serous cystadenocarcinoma.

Variable	*P* value	Hazard ratio	95%CI	Variable	*P* value	Hazard ratio	95%CI
Stage	0.467	1.480	0.514–4.266	Stage	0.483	1.457	0.509–4.176
Subdivision	0.371	1.242	0.772–1.998	Subdivision	0.493	1.181	0.733–1.904
Grade	0.096	2.003	0.883–4.544	Grade	0.055	2.216	0.984–4.98
MMP-19	0.006	1.079	1.023–1.139	MMP-20	0.003	1.660	1.185–2.324

^a^“MMP” (matrix metalloproteinase); “CI” (confidence interval).

^b^Data were analyzed using a multivariate cox model.

### High MMP-19 and -20 expressions induce drug resistance

**Figure 3:**
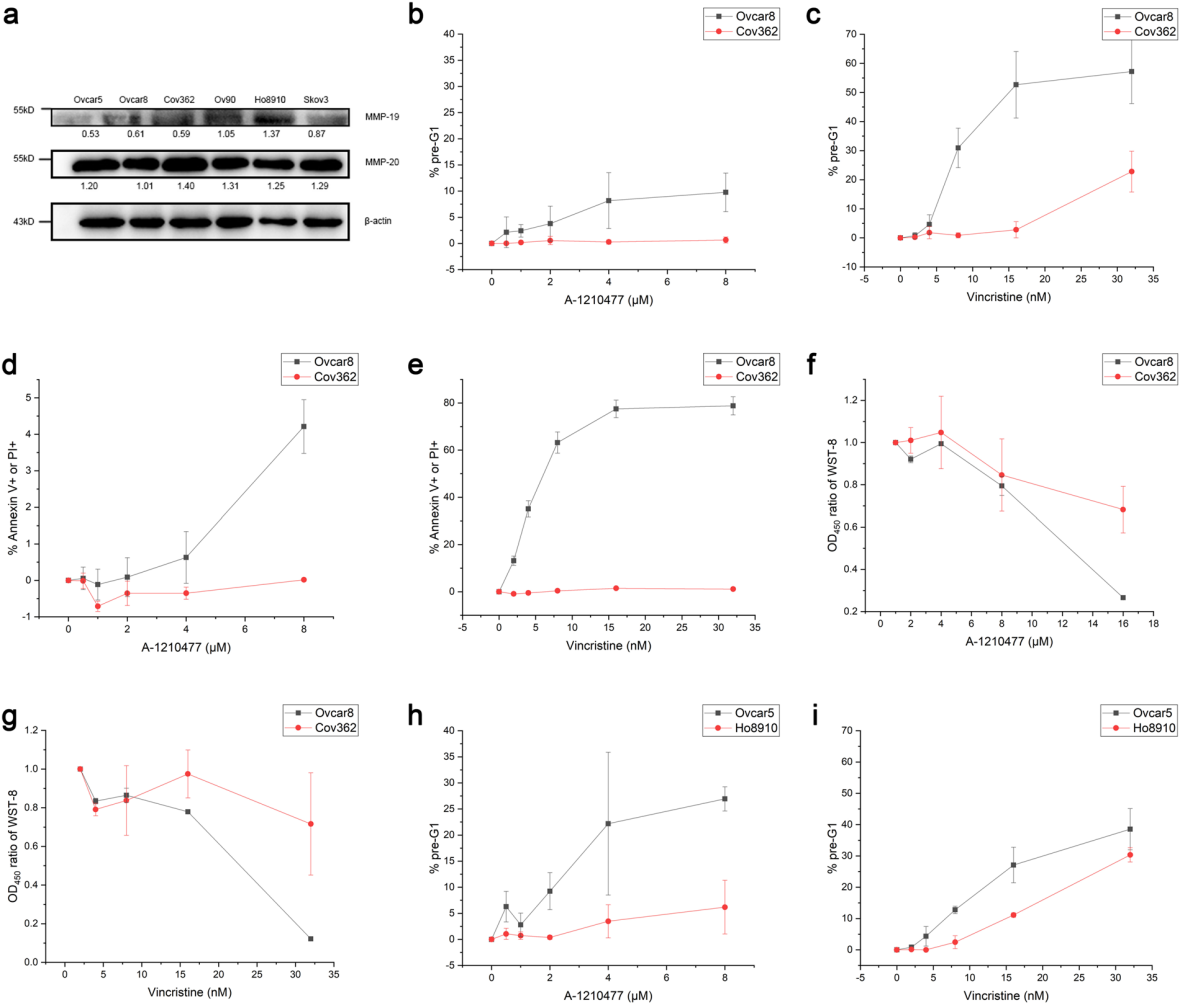
MMP-19 and MMP-20 high expressions induced anti-cancer drug resistance. (**a**) MMP-19 and MMP-20 were assayed by Western Blotting in indicated ovarian carcinoma cell lines. The numbers listed below the blots indicate the optical density ratio of target protein to β-actin. (**b–g**) Direct comparison of cell death and viabilities of ovarian cancer cell lines with low (Ovcar8) and high (Cov362) MMP-20 expressions after the treatment of A-1210477 (**b,d,f**) and Vincristine (**c,e,g**). After the cell lines were treated with indicated drugs for 48 hours, the percentages of pre-G1 cells (**b**,**c**), Annexin V+ or PI+ cells (**d,e**), or the ratio of OD450 by WST-8 (**f,g**) were assayed. (**h,i**) Direct comparison of cell death and viabilities of ovarian cancer cell lines with low (Ovcar5) and high (Ho8910) MMP-19 expressions after the treatment of A-1210477 (**h**) and Vincristine (**i**). After the cell lines were treated with indicated drugs for 48 hours, the percentages of pre-G1 cells were measured. Error bars, mean ± S.D. of three independent experiments.

Drug resistance^29^ and metastasis or invasion^15,16^ were the possible reasons of poor prognosis caused by high MMP expressions. We first test if drug resistance might be one of the reasons of the poor prognosis caused by high expressions of MMP-19 and MMP-20. MMP-19 and MMP-20 protein expression levels from six ovarian carcinoma cell lines (Ovcar5, Ovcar8, Cov362, Ov90, Ho8910 and Skov3) were assayed by western blotting. The MMP-19 and MMP-20 protein expression levels varied among these six cell lines, as indicated by optical density ratio of target protein to β-actin (Fig. 3a). Among these cell lines, MMP-19 protein level expressed highest in Ho8910 but lowest in Ovcar5, while MMP-20 expressed highest in Cov362 but lowest in Ovcar8. To further test the relationships of MMP-19 and MMP-20 protein levels and the anti-cancer drug sensitivities, both the MMP-19 and MMP-20 high expression and low expression cell lines were exposed to two different anti-cancer agents. One is A-1210477, which could direct target the anti-apoptotic protein MCL1 and induce apoptosis, and the other is Vincristine, which is a common chemotherapy drug used in many types of cancers, inducing cancer cell death through a microtubule polymerization mechanism. We observed that Ovcar8 (the lowest MMP-20 protein expression among the six cell lines), was more sensitive than Cov362 (highest MMP-20 expression among these lines) to both A-1210477 and Vincrinstine, as evaluated by the apoptotic pre-G1 cells (Fig. 3b,c) and also the annexin V and PI double staining assay (Fig. 3d,e). Moreover, less cell viabilities were observed in Ovcar8 than Cov362 cells after both A-1210477 and Vincrinstine treatments (Fig. 3f,g). Similarly, Ovcar5, which had lowest MMP-19 expression, was more sensitive to both A-1210477 and Vincristine than Ho8910, which had highest MMP-20 expression (Fig. 3h,i). Therefore, cell lines with higher MMP-19 or MMP-20 expression levels were associated with increased drug resistances to A-1210477 and Vincritine.

**Figure 4:**
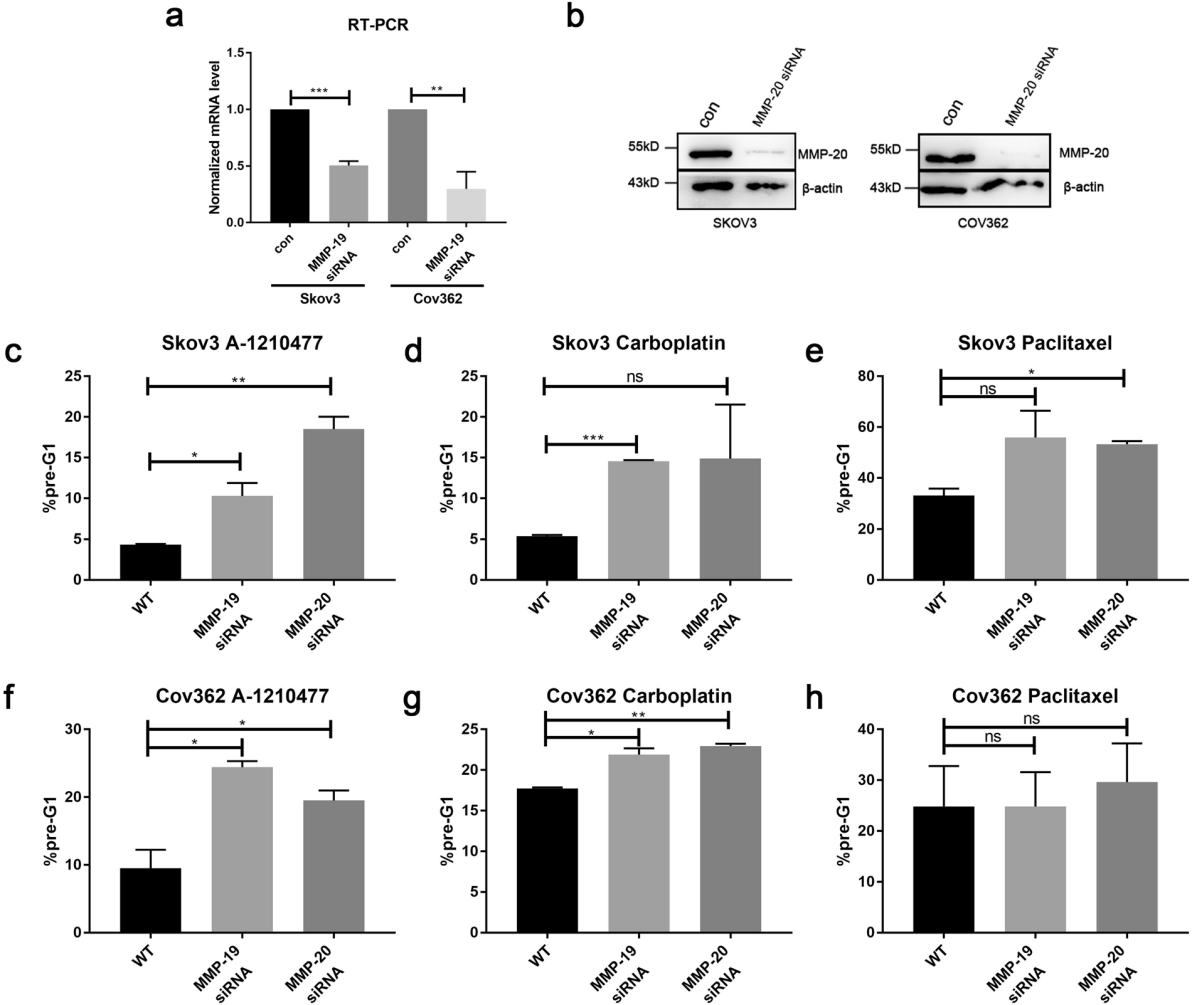
MMP-19 and MMP-20 knockdown increased anti-cancer drugs sensitivities. (**a**) After Skov3 and Cov362 cells were transfected with MMP-19 siRNA or control, the cells were harvested and the mRNA levels of MMP-19 were measured using quantitative PCR. (**b**) after the Skov3 and Cov362 cells were transfected with MMP-20 siRNA or control, the cells were collected and probed for MMP-20 by western blotting. (**c–e**) After Skov3 cells were transfected with MMP-19 or MMP-20 siRNAs or control followed by the treatment of A-1210477 (8 μM, **c**), Carboplatin (20 nM, **d**) or paclitaxel (40 nM, **e**), the percentage of pre-G1 cells were measured. (**f**–**h**) After Cov362 cells were transfected with MMP-19 or MMP-20 siRNAs or control followed by the treatment of A-1210477 (8 μM, **f**), Carboplatin (20 nM, **g**) or paclitaxel (40 nM, **h**), the percentage of pre-G1 cells were measured. Error bars, mean ± S.D. of three independent experiments. ns, not significant; *p < 0.05; **p < 0.01; ***p < 0.001.

To further confirm the correlation between MMP-19 and MMP-20 high expressions and drug resistance, ovarian cancer cell lines Skov3 and Cov362 were performed with the MMP-19 and MMP-20 knockdown using siRNAs, followed by treatments of several anti-cancer drugs. The mRNA expressions of MMP-19 in Skov3 and Cov362 cells decreased about 50% and 74% respectively after MMP-19 siRNA treatments (Fig. 4a). MMP-20 siRNA also remarkablely decreased the MMP-20 protein levels in both cell lines (Fig. 4b). More importantly, knockdown of MMP-19 significantly increased sensitivities of Skov3 to A-1210477 (p < 0.05) and Carboplatin (p < 0.001), as well as the sensitivities of Cov362 to A-1210477 (p < 0.05) and Carboplatin (p < 0.05) (Fig. 4c–h). Similarly, knockdown of MMP-20 significantly increased sensitivities of Skov3 to A-1210477 (p < 0.01) and Paclitaxel (p < 0.05), as well as sensitivities of Cov362 to A-1210477 (p < 0.05) and Carboplatin (p < 0.01) (Fig. 4c–h). To rule out the possibility of non-specific target effect of siRNAs, another siRNA was performed in Cov362 cell lines followed by drug treatments. As shown in Fig. S1a–d, MMP-19 siRNA#2 treatment significantly increased the sensitivities of Cov362 to A-1210477 (p < 0.05), Carboplatin (p < 0.01) and Paclitaxel (p < 0.001). Taken together, these results in Figs 3, 4 and S1 suggested high expression of MMP-19 and MMP-20 was associated with drug resistance in at least some types of ovarian cancer cells.

### High MMP-19 and -20 expressions promote invasiveness

**Figure 5:**
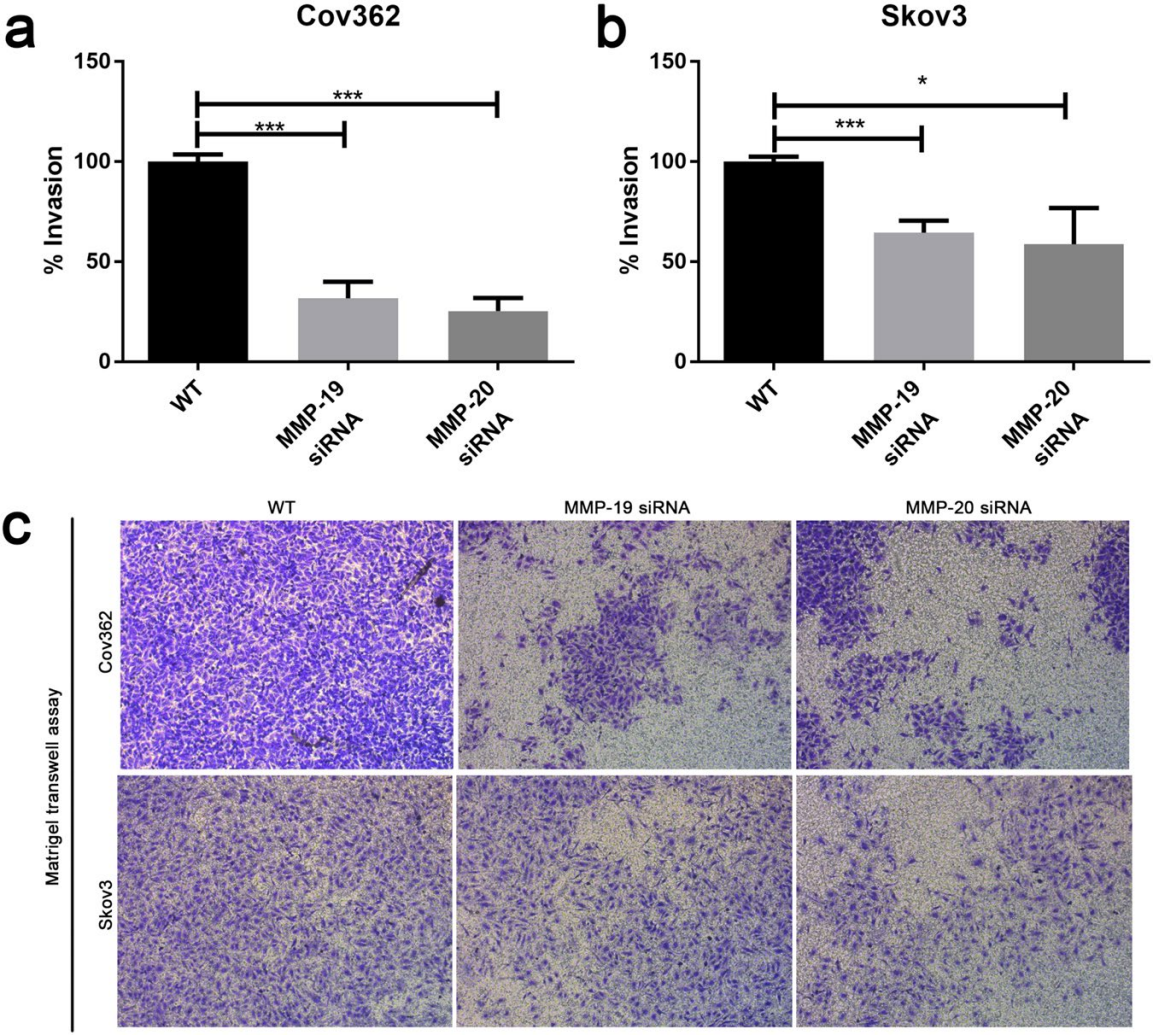
MMP-19 and MMP-20 knockdown decreased the invasion abilities of ovarian cancer cells. The normalized percentages of invaded Cov362 (**a**) and Skov3 (**b**) after transfected with MMP-19 siRNA or MMP-20 siRNA or control were indicated as detected by Boyden Chamber Transwell method. (**c**) A representative cell invasion assay was shown. Cells were transfected with MMP-19 or MMP-20 siRNA or control and incubated for 24 h, seeded into Matrigel-coated transwell inserts, and incubated for another 48 h. Error bars, mean ± S.D. of three independent experiments. *p < 0.05; **p < 0.01; ***p < 0.001.

Previous studies have also shown that MMPs are involved in cancer metastasis and invasion, so we also investigated the influence of MMP-19 and MMP-20 on cell invasion abilities using the Boyden chamber method. We observed that the MMP-19 and MMP-20 knockdown decreased Cov362 cell invasion abilities by 68% and 74%, respectively (Fig. 5a,c). Similar results were observed in ovarian cell line Skov3, that knockdown of MMP-19 and MMP-20 decreased the cell invasion abilities by 35% and 41%, respectively (Fig. 5b,c). In addition, another group of MMP-19 siRNA also decreased the Cov362 cell invasion ability by 52% (Fig. S2a,b), further confirmed the MMP-19 function in cell invasion ability. Therefore, the results in Figs 5 and S2 suggest high MMP-19 and -20 expressions related to high cancer cell invasion abilities.

## Discussion

Several previous studies have investigated the potential prognostic impact of one or more MMPs in different cancer types^13,15,21^, however, most of these studies only involve one or several MMP family members. No systematic study between MMPs and cancer prognosis has been done. In this study, we systematically investigated the relationship between MMP expressions and the patient clinicopathologic characteristics, and outcomes using clinical and gene expression data from TCGA of the ovarian cancer patients.

Through the analysis, we found the mRNA expression levels of certain MMP members were correlated with some clinic pathologic characteristics. For example, mRNA levels of MMP-3 and MMP-25 were significantly higher in stage III and IV tumors compared with stage II tumors (Table 2). Both MMPs have previously been reported to be associated with cancer metastasis or cancer progression. Between them, MMP-3 was reported to be overexpressed in both chicken and human ovarian cancers cells^30^, which would lead to the invasion of ovarian cancer^31^, and the molecular mechanisms might involve the miR-200 down regulation. MMP-25 was one of the membrane type MMPs, which could promote cell growth and migration because of their presence on the cell surface^32^. Our work revealed that MMP-3 and MMP-25 high expression levels were correlated with higher OSC tumor stage, providing further evidence of these MMPs in OSC progression.

By the analysis of the relationships between expressions of the different MMPs, multiple statistically significant strong or medium correlations were found between MMP pairs, which would suggest these pairs of MMPs were co-regulated by similar pathways, or even that one MMP is under the regulation of the other. In the present study, strong correlations (Table 3) were found between MMP-2 and MMP-11 (*P* < 0.001, *r* = 0.820), and MMP-11 and MMP-13 (*P* < 0.001, *r* = 0.805), suggesting strong co-regulations between both pairs of MMPs in OSC patients. Previous studies have also found correlations of both MMP pairs in breast cancer^28^. Moreover, we found medium correlations in quite a few pairs of MMPs. For example, medium correlations were found between MMP-2 and MMP-14 (*P* < 0.001, *r* = 0.764). A previous study has also shown that MMP-14 could activate MMP2, both of which appeared to play important roles in regulating cell growth and proliferation by controlling matrix remodeling in aggressive ovarian cancer cells^33^. We also found some of the statistically significant medium correlations that have not been reported before, including the correlations between MMP-1/MMP-3, MMP-1/MMP-13, MMP-2/MMP-3, and MMP-8/MMP-12. The reason why these MMPs showed significant correlations in OSC patient samples needs further study.

In this study, we also found high expressions of MMP-2, MMP-14, MMP-19, and MMP-20 were associated with poor overall survival using univariate analysis, however, only the high expression of MMP-19 and MMP-20 predict poor prognosis in a multivariate model, suggesting MMP-19 and MMP-20 high expressions as independent predictive factors for poor prognosis in OSC patients. Previous studies have also found that MMP-19 was highly expressed in astroglial tumors and facilitate the invasion of gliomacells^13^, and MMP-20 might play an important role in the progression of esophageal cancer^14^. In addition, we found that MMP-2 and MMP-14 correlated with poor prognosis in OSC patients using univariate analysis, both of which had been reported to predict poor prognosis in several cancer types^15,19,21,22^.

To further explore the potential mechanisms of MMP-19 and MMP-20 involved in poor prognosis, MMP related anti-cancer drug resistance and invasion mechanisms were studied using ovarian cancer cells lines. We found that ovarian cancer cell lines with higher MMP-19 and MMP-20 protein expressing levels were more resistant to anti-cancer drugs, such as A-1210477 and Vincristine (Fig. 3). While A-1210477 and Vincristine are not generally used in the treatment of OSC patient in clinic, we also included two other commonly used drugs to evaluate the relationship between drug resistance and the expressions of MMP-19 and MMP-20. One drug is Carboplatin, a DNA synthesis inhibitor, inhibits tumor growth by binding to DNA and interfering with DNA repair mechanisms, the other is Paclitaxel, a microtubule polymer stabilizer. MMP-19 and MMP-20 knockdown not only significantly increased the drug sensitivity to A-1210477 in both Skov3 and Cov362 cells, but also increased the drug sensitivity to Carboplatin in both cell lines, suggesting MMP-19 and MMP-20 high expression are related to at least some kind of anti-cancer drug resistances in OSC patients (Fig. 4). MMP-19 and MMP-20 knockdown did not increase Cov362 resistance to Paclitaxel (both p > 0.05, Fig. 4h), which might due to the fact that different molecular mechanisms are involved in the anti-cancer effects of these drugs.

Previous studies have also found high MMP expressions correlated with metastasis and invasion^13,15,16^. To further explore the mechanisms of OSC patients with poor prognosis and MMP-19 and MMP-20 high expression levels, we also conducted Boyden chamber assay to evaluate the ability of invasion after MMP-19 and MMP-20 knockdown. We found both MMP-19 and MMP-20 knockdown decreased the invasion ability in two OSC cell lines, Skov3 and Cov362 (Fig. 5). Taken together, our study suggested the potential mechanisms of poor prognosis of patients with MMP-19 and MMP-20 high expressions included both drug resistance and invasion caused by both MMPs.

In conclusion, our investigations of OSC patient samples from TCGA showed that high expression of MMP-19 and MMP-20 were independent predictors of poor outcome in patients with OSC. Moreover, through experiments using ovarian cancer cell lines, we found cell lines with high MMP-19 or MMP-20 expression levels were more resistant to several anti-cancer drugs. Further knockdown assay using MMP-19 and MMP-20 siRNAs confirmed the important roles of drug-resistance caused by MMP-19 and MMP-20 high expressions. In addition, we found MMP-19 and MMP-20 also increased cell invasion ability. All these *in vitro* studies provided potential mechanisms of the poor prognosis of OSC patient with high MMP-19 or MMP-20 RNA expressions.

## Methods

### Patients in TCGA

RNA-Seq expression and corresponding clinical data for OSC patients were downloaded from TCGA (http://cancergenome.nih.gov/). The methods of biospecimen procurement, RNA isolation, and RNA sequencing were previously described by the Cancer Genome Atlas Research Network^34^. RPKM (reads per kilobase per million mapped reads) was used as the expression value for statistical analysis.

### Statistical analysis

The relationships between MMP expression levels and the clinicpathological characteristics (stage, tumor grade, anatomic neoplasm subdivision) were analyzed by the Mann-Whitney U test. The Spearman correlation analysis was used to explore the relationships between expression levels of different MMPs. For patient outcome analysis, univariate and multivariate regression analyses were carried out. All of the MMPs expression levels were treated as continuous variables, which had the advantage of retaining all the data information as well as avoiding arbitrary cut-off points. Observed results for patient outcome analysis were described by hazard ratio with 95% confidence intervals (CIs). All *p* values were two-sided. MMPs that were statistically significant at the 0.05 level in the univariate analysis were again included in a multivariate regression analysis. All of the statistical analyses were performed using SPSS version 17.0.

### Antibodies and drugs

The following primary antibodies were used in this study: Goat polyclonal antibodies for MMP-19 (AF6790, R&D systems); Rabbit polyclonal antibodies for MMP-20 (ab198815, Abcam); Goat polyclonal antibodies for *β*-actin (Santa Cruz). The secondary antibodies were: HRP (horseradish peroxidase)-conjugate rabbit anti-goat IgG (abs20005–100 μl, Absin), HRP-conjugate anti-rabbit IgG (7076S, Cell Signaling Technology). A-1210477, Vincristine sulfate, Paclitaxel, and Carboplatin were from Medchemexpress.

### Cell culture

Ovarian carcinoma cell lines were kind gifts from Dr. Scott Kaufmann at Mayo Clinic. All cell lines were cultured in RPMI medium modified (Hyclone, SH30809.01) with 2.05 nM L-glutamine, 1% streptomycin-penicillin (Hyclone, SV30010, 10000 units/ml penicillin and 10000 μg/ml streptomycin), and 10% fetal bovine serum (Clark, FB25015).

### Drug sensitivity assays

For pre-G1 analysis, log-phase cells were treated for 48 h (A-1210477 or Vincristine), washed twice with PBS, permeabilized with 0.1% Triton X-100 and stained with Propidium Iodide. The apoptotic cells were evaluated as the pre-G1 proportions in the cell cycle. After 20,000 events were collected on a Beckman coulter flow cytometer, data were analyzed using CytExpert1.2 software.

Alternatively, for Annxin V and PI double staining assay, the cells were washed twice with PBS after harvest, and stained with Annxin V and PI in the buffer containing 10 mM HEPES, 140 mM NaCl, 2.5 mM CaCl_2_, pH 7.4.

### Western blotting

After 1 × 10^7^ cells were collected and washed with ice-cold PBS, cells were lysed in 500 μl ice cold RIPA buffer, supplemented with protease inhibitor cocoktail (Roche), then disrupted five times on a sonicator on ice under 30% strength. The Cell lysates were further incubated 30 min on ice. After centrifugation for 15 min at 13,000 g, the supernants were subjected to SDS-PAGE and Western Blotted for MMP-19, MMP-20.

### SiRNAs and cell transfection

The sequences of MMP-19 and MMP-20 siRNAs were as following: MMP-19 siRNA #1 (5′-GCCUAGAGGAUCCCUUCAATT-3′), MMP-19 siRNA #2 (5′-GCAGCUUCGAGUAGAGAAATT-3′), and MMP-20 siRNA (5′-UCCUUUGACGCUGUGACAATT-3′). Cells were transfected with siRNAs at equal volume using siRNA-Mate plus (GenePharma, G04003) following the manufactor’s instructions.

### Invasion assays

Transwell chambers with 8-μm pores (Corning; NY, USA) were used. For the tumor cell invasion assay, the transwell membrane was pre-coated with 50 μl of Matrigel matrix (1:8 mixed with RPMI 1640; BioFroxx, Cat NO.1567ML005). Cell suspension in serum-free medium was added to the upper chamber, and then incubated in 5% CO2 for 48 h. After incubation, invaded cells were fixed with methanol for 30 min, stained with 0.1% crystal violet, and counted under a light microscope at 100× magnification (Olympus, CKX3-SLP; JAPAN).

## Supplementary information

Supplemental Information

## Data Availability

The datasets generated and analysed during the current study are available from the corresponding author on reasonable request.
