# Supplementary material for: Matrix Metalloproteinase Expressions Play Important role in Prediction of Ovarian Cancer Outcome
Source: Sci Rep. 2019 Aug 12;9:11677. doi: 10.1038/s41598-019-47871-5 (PMC6691000; doi:10.1038/s41598-019-47871-5)
Supplement: Supplementary file 1 — Supplemental Information [file 41598_2019_47871_MOESM1_ESM.pdf]

Matrix Metalloproteinase Expressions Play Important role in Prediction of Ovarian Cancer Outcome

Shujie Wang[1,2,+], Jia Jia[1,2,+], Dongyan Liu[1,2], Meng Wang[3], Zhen Wang[3], Xueling Li[1,2], Hongzhi Wang[1,2], Yong Rui[2], Zhirong Liu[2], Wei Guo[1,2], Jinfu Nie[1,2,3*] and Haiming Dai[1,2,*]

[1]Anhui Province Key Laboratory of Medical Physics and Technology, Center of Medical Physics and Technology, Hefei Institutes of Physical Science, Chinese Academy of Sciences, Hefei, Anhui, China

[2]Hefei Cancer Hospital, Chinese Academy of Sciences, Hefei, Anhui, China

[3]Hefei Institute of Stem Cell and Regenerative Medicine, Guangzhou Institutes of Biomedicine and Health, Chinese Academy of Sciences, China

* Corresponding authors: Daih@cmpt.ac.cn or jinfunie@163.com

[+]these authors contributed equally to this work

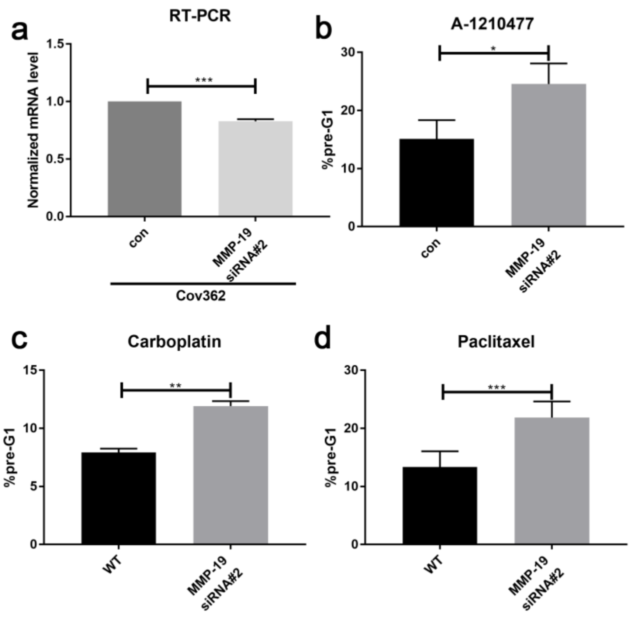

Supplemental Figure 1. Another MMP-19 siRNA#2 knockdown increased anti-cancer drugs sensitivities. a, After Cov362 cells were transfected with MMP-19 siRNA#2 or control, the cells were harvested and the mRNA levels of MMP-19 were measured using quantitative PCR. (b-d) After Cov362 cells were transfected with MMP-19 siRNA#2 or control followed by the treatment of A-1210477 (8μM, b), Carboplatin (20 nM, c) or paclitaxel (40 nM, d), the percentage of pre-G1 cells were measured. *, $p<0.05$; **, $p<0.01$; ***, $p<0.001$.

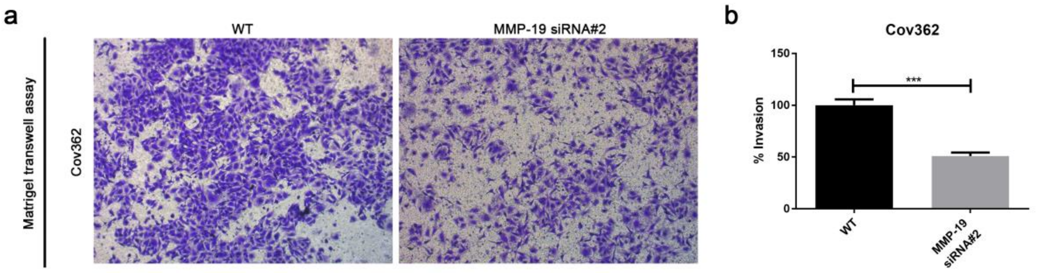

Supplemental Figure 2. Another MMP-19 siRNA#2 knockdown decreased the invasion abilities of ovarian cancer cells. A representative cell invasion assay (a) and the normalized percentages of invaded Cov362 (b) after transfected with MMP-19 siRNA or control were indicated as detected by Boyden Chamber Transwell method. Error bars, mean ± S.D. of three independent experiments. ***, $p<0.001$.

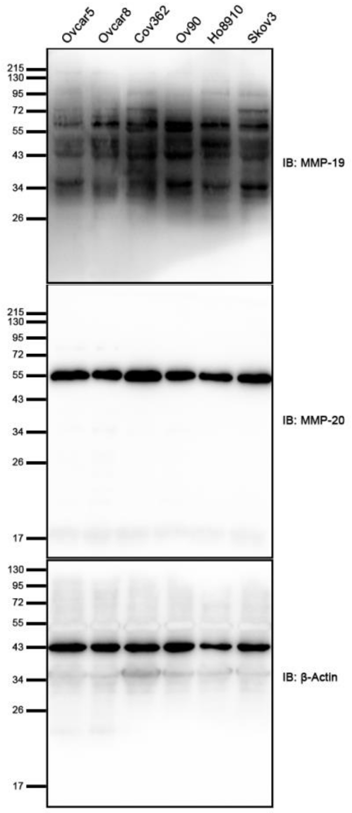

Supplemental Figure 3. Original figures of western blots in Figure 3(a).
